# Genetic and Lifestyle Risks for Coronary Artery Disease and Long-Term Risk of Incident Dementia Subtypes

**DOI:** 10.1161/CIRCULATIONAHA.124.070632

**Published:** 2025-04-04

**Authors:** Arisa Sittichokkananon, Victoria Garfield, Scott T. Chiesa

**Affiliations:** Princess Srisavangavadhana College of Medicine, Chulabhorn Royal Academy, Bangkok, Thailand (A.S.).; Department of Pharmacology & Therapeutics, Institute of Systems, Molecular, & Integrative Biology, University of Liverpool, UK (V.G.).; Department of Population Science and Experimental Medicine, Institute of Cardiovascular Science, UCL, London, UK (A.S., S.T.C.).

**Keywords:** Alzheimer’s disease, dementia, dementia, vascular, heart disease risk factors

## Abstract

**BACKGROUND:**

Shared genetic and lifestyle risk factors may underlie the development of both coronary artery disease (CAD) and dementia. We examined whether an increased genetic risk for CAD is associated with long-term risk of developing all-cause, Alzheimer’s, or vascular dementia, and investigated whether differences in potentially modifiable lifestyle factors in the mid- to late-life period may attenuate this risk.

**METHODS:**

A prospective cohort study of 365 782 participants free from dementia for at least 5 years after baseline assessment was conducted within the UK Biobank cohort. Genetic risk was assessed using a genomewide polygenic risk score (PRS) for CAD and lifestyle risk using a modified version of the American Heart Association’s Life’s Essential 8 Lifestyle Risk Score (LRS). Higher values for both scores were deemed to represent increased risk. Primary outcomes were incident all-cause, Alzheimer’s, and vascular dementia diagnoses obtained from self-report and electronic health records. Secondary outcomes were neuroimaging phenotypes measured in 32 028 participants recalled for magnetic resonance imaging. Sensitivity analyses were conducted to test the extent by which biological and behavioral risk factors contributed to observed associations.

**RESULTS:**

A total of 8870 cases of all-cause dementia were observed over a median 13.9-year follow-up. Both genetic (PRS) and lifestyle (LRS) risk scores for CAD were associated with a modestly elevated risk of all-cause dementia (subhazard ratio per SD increase, 1.10 [1.08, 1.12], *P*<0.001, for PRS and 1.04 [1.02, 1.06], *P*=0.006, for LRS). This risk appeared largely attributable to underlying vascular dementia diagnoses (subhazard ratio, 1.16 [1.11, 1.21], *P*<0.001 for PRS and 1.15 [1.09, 1.22], *P*<0.001, for LRS), because Alzheimer’s disease was found to demonstrate moderate associations with PRS alone (subhazard ratio, 1.09 [1.06, 1.13]; *P*<0.001). LRS was found to have an additive rather than interactive effect with PRS, with individuals in the highest tertiles for both genetic and lifestyle risk for CAD ≈70% more likely to develop vascular dementia during follow-up compared with those in the lowest tertiles for both (subhazard ratio, 1.71 [1.39, 2.11]; *P*<0.001). This was substantially attenuated in those with a low LRS at baseline, however, regardless of underlying genetic risk (30% reduction for low versus high LRS tertile regardless of PRS tertile; *P*<0.001 for all). In a subset of individuals recalled for neuroimaging assessments, those in the highest tertiles for genetic and lifestyle risk for CAD demonstrated a ≈25% greater volume of white matter hyperintensities than those in the lowest risk tertiles, but showed little difference in gray matter or hippocampal volumes. Sensitivity analyses identified associations between both biological and behavioral risk scores with white matter hyperintensity burden and vascular dementia, whereas some Alzheimer’s dementia associations showed seemingly paradoxical relationships.

**CONCLUSIONS:**

Individuals who are genetically predisposed to developing CAD also face an increased risk of developing dementia in old age. This risk is reduced in those demonstrating healthy lifestyle profiles earlier in the lifespan, particularly in those who may be at an increased risk of developing dementia caused by an underlying vascular pathology.

**Clinical Perspective**

**What Is New?**

- Individuals with coronary artery disease are also at increased risk for developing dementia. Recent attempts to causally link the presence of established coronary artery disease to future dementia risk have proved unsuccessful, suggesting that shared pathogenic pathways common to both diseases may instead explain this relationship.
- Underlying genetic risk for coronary artery disease has previously been associated with reduced cognitive function and brain volumes in later life, but no long-term follow-up study has been performed to investigate its relationship with future diagnoses of different subtypes of dementia, or the extent by which healthy lifestyle behaviors may modify these relationships.

**What Are the Clinical Implications?**

- Our findings suggest that an underlying genetic risk for coronary artery disease is associated with both an increased risk of cerebrovascular damage in midlife to late life and an increased risk of vascular dementia in long-term follow-up.
- Healthy lifestyle behaviors prior to or during the mid- to late-life period may attenuate this risk regardless of genetic predisposition, particularly in individuals who may be at risk of developing dementia caused by an underlying vascular pathology.
- Lifecourse prevention strategies aimed at reducing the population burden of cardiovascular disease may also protect against progression to vascular dementia in older age.

Coronary artery disease (CAD) and dementia are 2 of the leading causes of death and disability worldwide, accounting for an estimated annual burden of 9 million and 2 million deaths, respectively.^1,2^ Both conditions occur as the result of separate and complex disease processes arising from diverse pathogeneses, but accumulating evidence suggests that each may share common, and at times potentially modifiable, underlying risk factors that act to simultaneously increase the risk of both diseases.

The most common cause of dementia is Alzheimer’s disease (AD), which is responsible for 60% to 70% of dementia diagnoses.^3^ AD is characterized by a progressive decline in memory and thinking skills believed to arise from an accumulation of amyloid plaques and neurofibrillary tau tangles in the brain, which are the hallmark of a diagnosis. Recent years have seen a wealth of research investigating the role that genetic variants play in the development of AD,^4–6^ and how this risk may be attenuated by the adoption of healthy lifestyle behaviors at a younger age.^7–10^

Cerebrovascular disease also contributes to a large proportion of dementia diagnoses, both in the form of overt vascular dementia and as a copathology present in an estimated 50% to 80% of AD diagnoses.^11–13^ Because atherosclerosis-related hypoperfusion plays a major role in both CAD and cerebrovascular disease,^14,15^ an underlying genetic predisposition to the development of atherosclerosis across the life course may therefore represent an important link in the relationship between heart and brain health. A number of previous studies have sought, and broadly failed, to establish a causal relationship linking manifest CAD to dementia risk using instrumental variable study designs, such as Mendelian randomization.^16,17^ However, no study to date has investigated the potential for shared underlying genetic pathways to simultaneously contribute to the pathogenesis of both diseases or investigated the impact that healthy lifestyle behaviors earlier in the life span may have on different dementia outcomes.

Using data from a large-scale cohort study of >360 000 participants prospectively followed for a median of 14 years, our primary aims were to assess: whether a genomewide polygenic risk score (PRS) for CAD was associated with an increased risk of developing all-cause dementia, AD, or vascular dementia during the transition through midlife to late life; whether this risk was attenuated in those rated as having low lifestyle risk scores at their baseline assessment; and whether any potential associations with lifestyle risk were more likely to be explained by differences in underlying biological or behavioral risk factors. A secondary aim was to assess underlying changes in brain structure that may explain these differences in risk in a subset of more than ≈32 000 individuals recalled for magnetic resonance neuroimaging.

## Methods

### Data Availability

All data used in this publication are open access and available to bona fide researchers through well-documented processes detailed at https://www.ukbiobank.ac.uk. The statistical code for all analyses in this article can be found in an open-access GitHub repository located at https://github.com/scottchiesa/UKB_PRS_LRS_Dementia.

### Study Population

This study used data from the UK Biobank, a large population-based cohort of >500 000 individuals recruited in the United Kingdom from 2006 through 2010. The UK Biobank study received ethical approval from the National Health Service North-West Multicentre research ethics committee. All participants gave informed consent during the baseline assessment. Any participants withdrawing consent before commencement of this study were excluded before analysis. Participants were also excluded if they had prevalent dementia (ie, a diagnosis of dementia before baseline), were diagnosed with dementia within 5 years of baseline (to minimize risk of undiagnosed dementia cases), or were younger than 50 years of age at baseline (because the primary outcome of interest in this study was late-onset dementia, which occurs after 65 years of age). Participants with non-White ancestry were also excluded to minimize residual confounding through underlying ancestral differences and possible population stratification.^18^

### Exposures

#### Polygenic Risk Score

This study used the standard UK Biobank PRS for CAD derived by Thompson et al,^19^ full details of which are described therein. In brief, a Bayesian approach was used to generate the CAD PRS from a meta-analysis of genomewide association study summary statistics using multiple external studies. All analyses included age at assessment, sex, genotyping chip, and 10 principal components as covariates. Per-individual PRS values were calculated as the genomewide sum of the per-variant posterior effect size multiplied by allele dosage. To avoid the risk of overfitting, the PRS was developed only on the basis of non–UK Biobank populations before being calculated for all individuals in UK Biobank.

#### Lifestyle Risk Score

A lifestyle risk score (LRS) was next created based on an adapted form of the American Heart Association Life’s Essential 8 (LE8) concept, created to measure and monitor cardiovascular health. LE8 consists of 4 biological (body mass index [BMI], blood lipid level, blood glucose level, and blood pressure [BP]) and 4 behavioral (diet, physical activity, smoking status, and sleep duration) components.^20^ Physical activity, smoking status, sleep quality, and diet were assessed at the initial assessment center visit using a touchscreen questionnaire. Participants were asked whether they currently smoke or if they have ever smoked in the past. They were also asked about the frequency, duration, and intensity of their physical activities. Sleep quality was assessed by self-reporting how many hours of sleep they got in every 24-hour period. For diet, participants answered questions about how many servings of fruit, vegetables, whole grains, and refined grains they had each day, and how many servings of processed meats, unprocessed red meats, and fish they had each week. BMI values were calculated as weight (kg)/height (m)2 from height and weight measurements taken at the baseline visit. BP was reported as the average of 2 automated measures taken a few moments apart using an Omron device at baseline visit. For biomarkers, blood samples were collected from participants at each assessment center, refrigerated, and transported to a central laboratory for automated processing. Low-density lipoprotein cholesterol level was measured by enzymatic protective selection analysis on a Beckman Coulter AU580. Hemoglobin A1c was used in place of fasting glucose to provide a better long-term marker of glycemic status and was measured using high-performance liquid chromatography analysis on a Bio-Rad VARIANT II Turbo. Extreme values ±4 SD from the mean were excluded from the LRS calculation. Whereas LE8 is usually studied as a positive measure, with high values possibly having protective effects, here it was flipped to be used as a measure of lifestyle risk in the same manner as the PRS. Participants were scored 0 to 2 points for each lifestyle component, with higher scores representing greater cardiovascular risk (Table 1). The points from each lifestyle category were then summed to give an overall risk score of 0 to 16, with higher numbers again indicating greater risk.

**Table 1.**
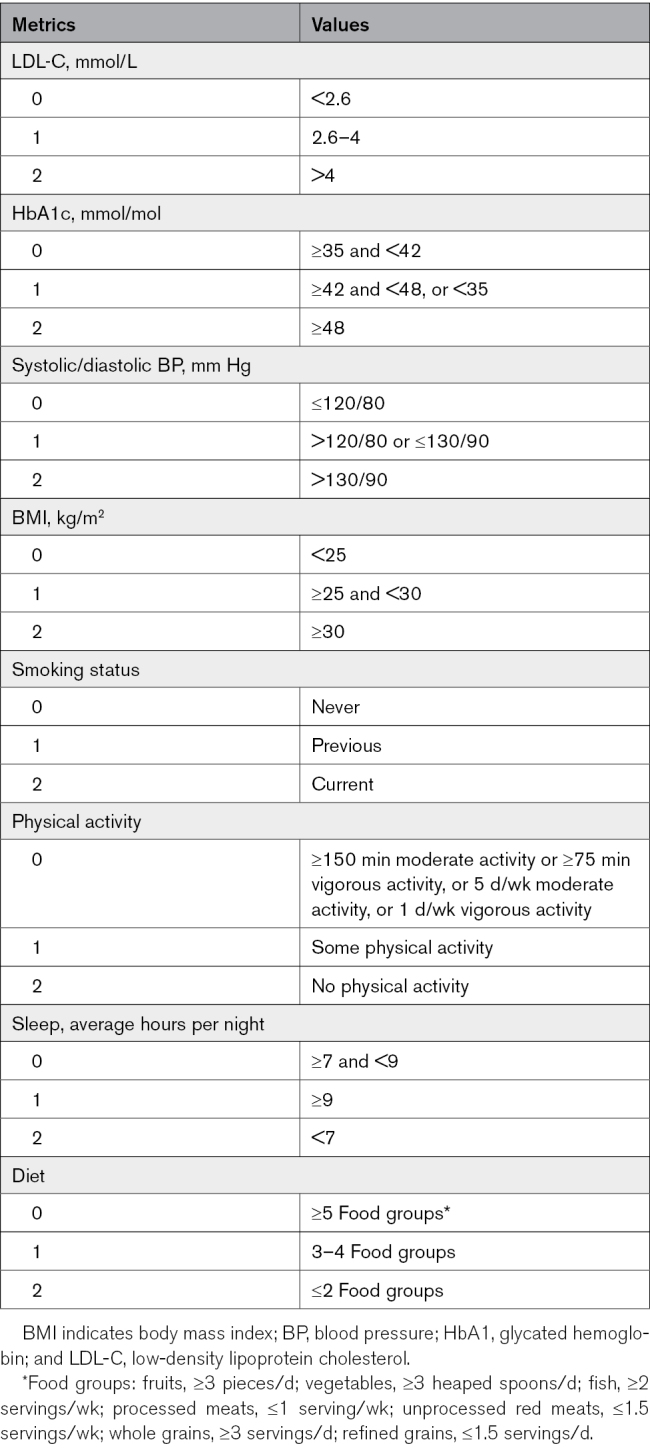
Cardiovascular Health Metrics

### Outcomes

#### Dementia Diagnoses

Incident dementia was captured using the algorithmic method detailed by Wilkinson et al,^21^ which used data from both participant self-report and linked UK hospital admission and mortality records. *International Classification of Diseases* (9th and 10th editions) codes were used to identify dementia cases in linked hospital episode statistics data. Three subtypes of dementia were included in this analysis: all-cause dementia, AD, and vascular dementia.

#### Neuroimaging Phenotypes

Three common neuroimaging phenotypes with well-established links to future risk of dementia were chosen as imaging outcomes in secondary analyses: cortical gray matter volume,^22^ hippocampal volume,^23^ and white matter hyperintensity (WMH) volume.^24^ Full details on the imaging-derived phenotypes available in the UK Biobank can be found elsewhere.^25^ In brief, gray matter volume was extracted from T1-weighted images using FAST (FMRIB Automated Segmentation Tool; version 4.1) and hippocampal volume using FIRST (FMRIB Integrated Registration and Segmentation Tool; version 5.0). WMH volumes were calculated on the basis of T1- and T2-weighted fluid-attenuated inversion recovery and derived using BIANCA (Brain Intensity Abnormality Classification Algorithm).^26^

### Covariates

Covariates included age, sex, level of education completed (less than secondary, secondary, or higher), socioeconomic status (Townsend deprivation index), and self-reported use of lipid-, BP-, or glucose-lowering medications at baseline visit. Age was included as a covariate because dementia prevalence increases substantially with age.^27^ Sex was considered as a covariate because the ratio of male to female prevalence is different in each dementia subtype, with AD being more common in women and vascular dementia being more prevalent in men.^28^ Increased educational attainment is associated with reduced risk of both CAD risk factors and dementia,^29^ whereas lower socioeconomic status is associated with a greater risk.^30^ For neuroimaging analyses, gray matter and hippocampal volumes were additionally adjusted for total brain volume and childhood body size because of the known risk of confounding from these factors.^31^

### Statistical Analyses

Descriptive characteristics of the cohort were summarized by dementia status using percentages for categorical variables, mean and SD for normally distributed continuous variables, and median and interquartile range for non-normally distributed variables. Normality was assessed through visual inspection of histograms. Although technically an ordinal variable, LRS was found to be approximately normally distributed and was therefore *z* scored and treated as a continuous measure in all models to enable comparability with PRS analyses.

#### Dementia Diagnoses

Competing risks regressions based on Fine and Gray proportional subhazard models were used to examine associations between PRS and LRS as exposures and all-cause dementia, AD, and vascular dementia as outcomes. Death from any cause other than dementia was included in all models as a competing risk. Interactions between PRS and LRS were tested, as were interactions between both exposures and sex. Because the latter showed some evidence for a modifying effect on multiple outcomes, all models were run with sex included as a stratified variable, providing equal coefficients across strata in the pooled data while allowing a distinct baseline hazard for each sex. Time scale of follow-up was time since baseline assessment (2006–2010) until December 13, 2022. The proportionality of hazards assumption for each model was assessed using the Schoenfeld residuals technique.^32^ For PRS exposures, statistical models were adjusted for age and sex alone. For LRS exposures, models were additionally adjusted for highest education level, socioeconomic status, and lipid-, BP-, and glucose-lowering medications because of their known associations with lifestyle health factors. The decision not to adjust PRS models for these additional covariates was taken to avoid overadjustment or potential collider bias because of the possibility that these factors may lie on the causal pathway linking genetic risk for CAD to dementia development and thus may represent effect mediators rather than confounders. To examine incident dementia risk according to combined genetic and lifestyle risk, PRS and LRS were each stratified into categories before being combined for analyses. PRS was split into 3 equal tertiles, in which 33.3% of participants had low, intermediate, or high genetic risk, respectively. LRS was also split into 3 approximately equal groups, with 34.4% of participants categorized as having low lifestyle risk (ie, scoring <6 points), 35.4% having intermediate lifestyle risk (ie, scoring 6 or 7 points), and 30.2% having high lifestyle risk (ie, scoring 8–16 points). Similar models to those described were then performed on the 9 combined genetic and lifestyle risk categories, with combined low genetic and lifestyle risk as the reference group.

#### Neuroimaging Phenotypes

For neuroimaging measures, multivariable linear regression models were used to test associations between all of the exposures listed previously and 3 structural neuroimaging outcomes (cortical gray matter volume, hippocampal volume, and WMH volume). For ease of comparison, all outcomes were log-transformed before inclusion in models before being back-transformed for reporting and are therefore expressed as a percentage change in geometric mean per unit change in exposure. PRS analyses were adjusted for age, sex, total brain volume (the latter not for WMHs), and childhood body size; LRS analyses were adjusted for age, sex, highest education level, current socioeconomic status, baseline medication use, total brain volume (again not for WMHs), and childhood body size.

#### Statistical Interpretation and Sensitivity Analyses

All analyses were performed in Stata version 15 (StataCorp LLC). Multiple imputation using chained equations (10 imputations) was used to account for missing data, and 3 sensitivity analyses were additionally run for comparison with the main results: PRS and LRS models were rerun using complete case analysis; PRS and LRS models were rerun after exclusion of participants with evidence of an opposing subtype of dementia (eg, removing people with a known vascular dementia diagnosis in analyses comparing people with and without an Alzheimer’s dementia diagnosis); and PRS and LRS models were rerun after further stratification of the LRS into biological (BMI, BP, lipid level, and blood glucose level) and behavioral (diet, physical activity, sleep, and smoking) subscores. All models in sensitivity analyses were identical to those used in primary analyses, with one additional model adjusting for lifestyle behaviors also included when assessing the biological risk score to account for potential behavioral confounding. An a priori decision was made to interpret findings mainly on the basis of model estimates and their 95% CIs rather than assigning significance using an arbitrary *P* value cutoff of 0.05.^33^ These *P* values are still highlighted throughout for reference.

## Results

### Sample Characteristics

**Figure 1.**
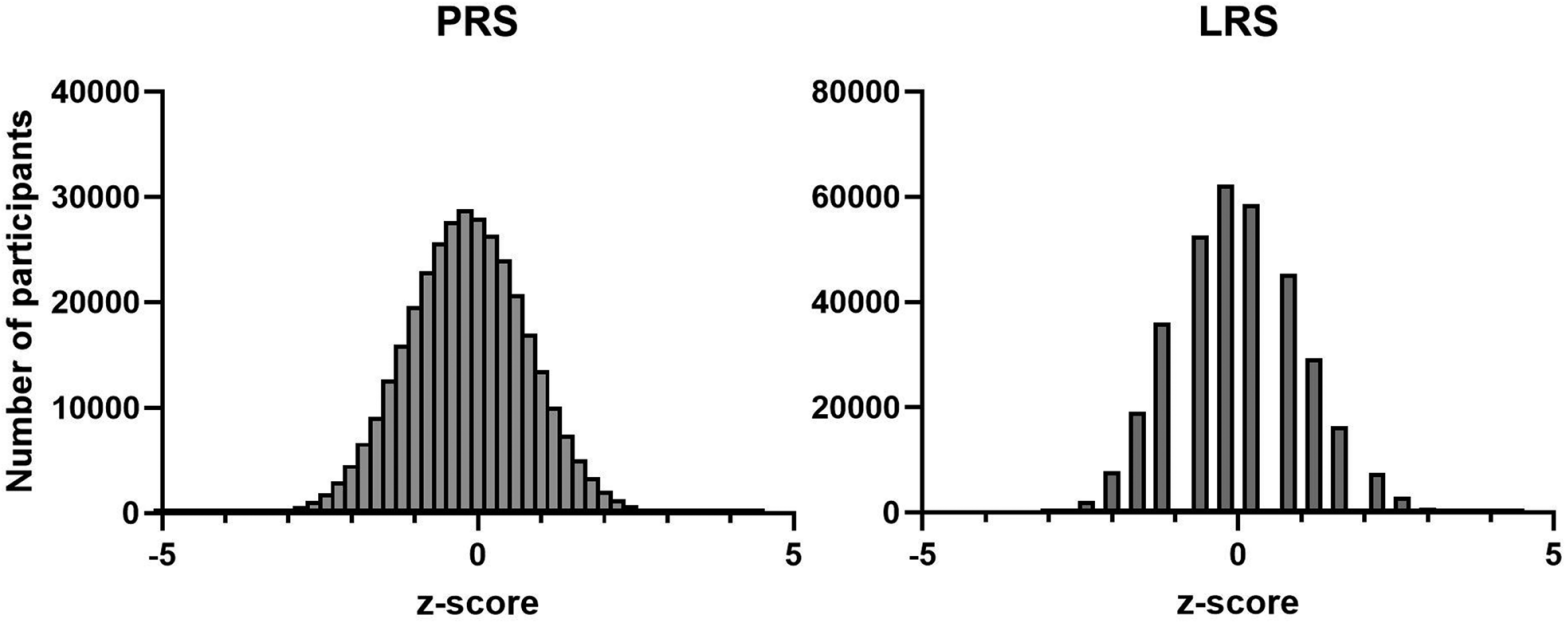
**Distribution of polygenic and lifestyle risk scores for coronary artery disease.** LRS indicates lifestyle risk score; and PRS, polygenic risk score.

The final study sample consisted of 365 782 individuals, 8870 of whom had all-cause dementia during a median follow-up of 13.9 years (Figure S1). Both PRS and LRS were approximately normally distributed (Figure 1). Participants with dementia were on average older at baseline than participants without dementia. A greater proportion of participants with AD were female, whereas participants with vascular dementia were more likely to be male. Full baseline characteristics are presented in Table 2. Details on the proportion of missing data that required imputation can be found in Table S1.

**Table 2.**
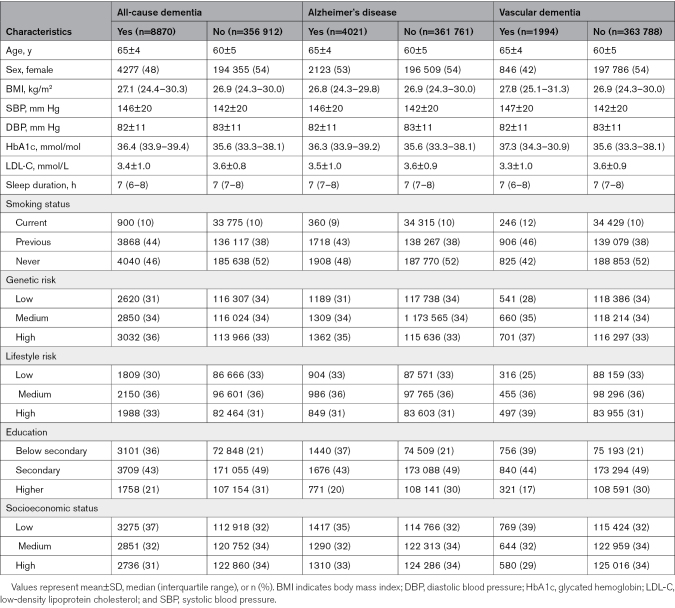
Participant Baseline Characteristics

### Genetic Risk for CAD and Future Dementia Risk

Higher genetic risk for CAD was associated with increased risk of developing all dementia types, both when expressed as a continuous variable and when stratified by tertiles (Table 3). This risk appeared to be greatest for vascular dementia, with those in the top tertile for CAD PRS showing nearly double the risk for a vascular diagnosis of dementia compared with AD (≈33% versus 18%, respectively; Table 3).

**Table 3.**
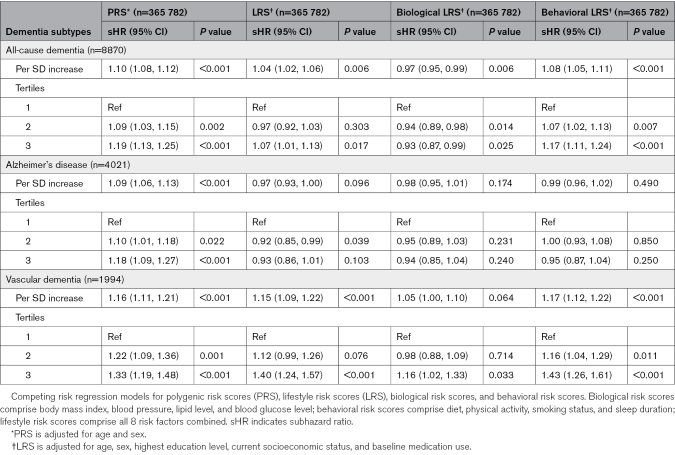
Associations Between Genetic and Lifestyle Risk for Coronary Artery Disease and Risk of Incident Dementia Subtypes

### Lifestyle Risk for CAD and Future Dementia Risk

Higher lifestyle risk for CAD was associated with a modestly increased risk of developing all-cause dementia (subhazard ratio [sHR; 95% CI] for top tertile, 1.07 [1.01, 1.13]; *P*=0.017; Table 3). This association appeared to be driven almost exclusively by vascular dementia, with those in the top tertile for CAD LRS having a 40% increased risk in fully adjusted models compared with those in the lowest tertile (sHR, 1.40 [1.24, 1.57]; *P*<0.001; Table 3), whereas no association was seen for AD (sHR, 0.93 [0.86, 1.01]; *P*=0.103; Table 3).

### Combined Genetic and Lifestyle Risk for CAD and Future Dementia Risk

**Figure 2.**
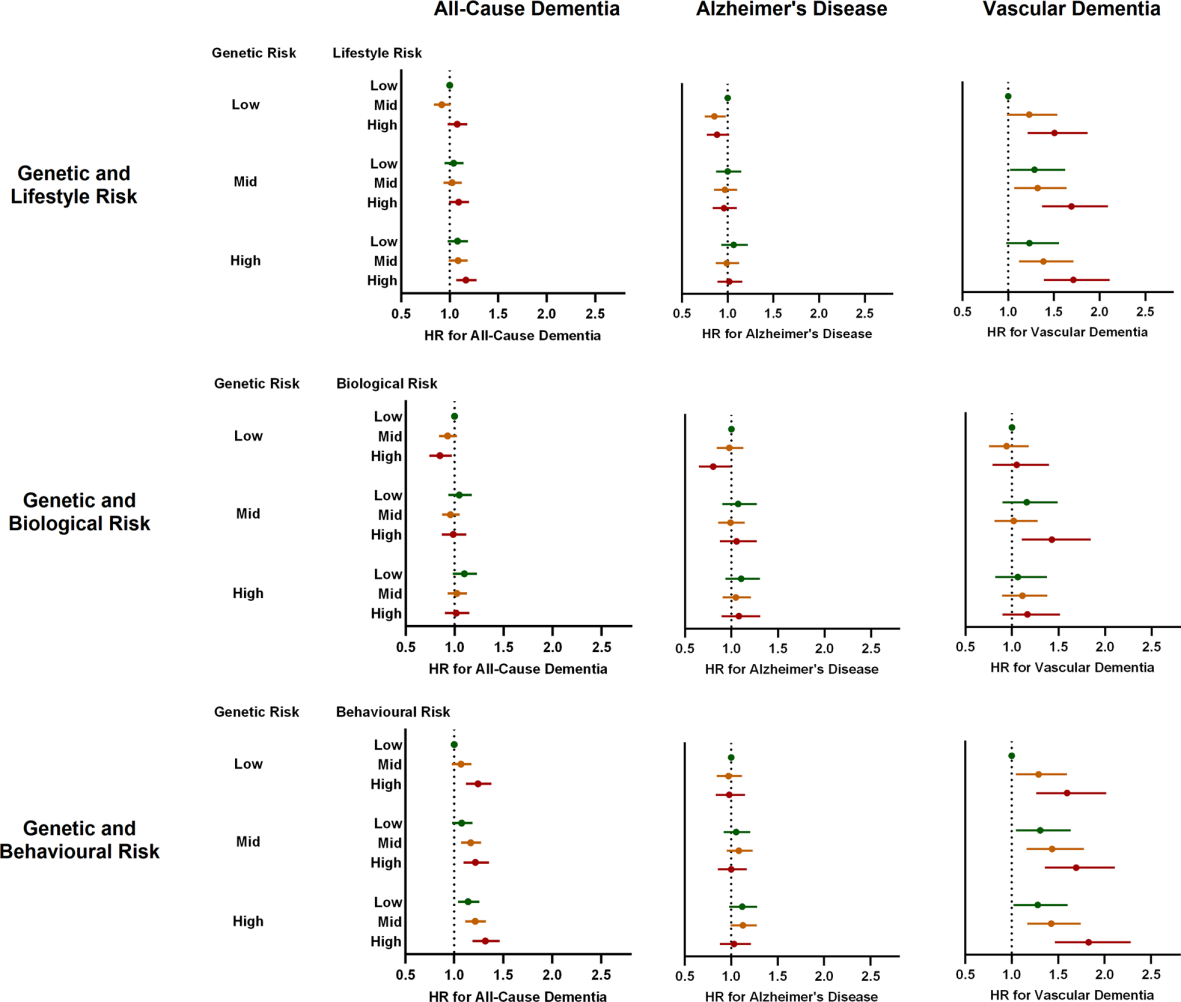
**Forest plots showing subhazard ratios for combined genetic and lifestyle risk for coronary artery disease and incident dementia subtypes.** Competing risk regression models adjusted for age, sex, highest education level, socioeconomic status, and baseline medication use. Data are expressed as subhazard ratio and 95% CI compared with the reference category of combined low genetic and lifestyle risk. Low, medium, and high categories of genetic risk refer to tertiles of polygenic risk score. Lifestyle risk refers to categories of an overall lifestyle risk score comprising body mass index, blood pressure, lipid level, blood glucose level, diet, physical activity, smoking status, and sleep duration. Biological risk refers to categories of a risk score comprising body mass index, blood pressure, lipid level, and blood glucose level. Behavioral risk refers to categories of a risk score comprising diet, physical activity, smoking status, and sleep duration. HR indicates subhazard ratio.

There was little evidence for an interaction between PRS and LRS for all-cause dementia (*P*=0.383), AD (*P*=0.917), or vascular dementia (*P*=0.793), suggesting that the association between lifestyle risk factors and future dementia risk did not vary depending on an individual’s underlying genetic risk. However, a combination of high genetic and lifestyle risk was found to have an additive effect on future dementia risk (Figure 2). This once again appeared predominantly attributable to associations between CAD risk factors and vascular dementia, with individuals in the highest tertiles for both PRS and LRS found to have a 71% higher risk of developing vascular dementia during long-term follow-up than those in the lowest tertiles (sHR, 1.71 [1.39–2.11]; *P*<0.001; Figure 2). Conversely, a low LRS was found to be associated with a reduced risk of vascular dementia regardless of underlying genetic risk (30% reduction for low versus high LRS tertile regardless of PRS tertile; *P*<0.001 for all; Figure 2). Little evidence was found for any additive effect of genetic and lifestyle risks for CAD on AD (sHR, 1.02 [0.89–1.16]; *P*=0.741; Figure 2).

### Genetic and Lifestyle Risk for CAD and Differences in Subclinical Neuroimaging Phenotypes

**Figure 3.**
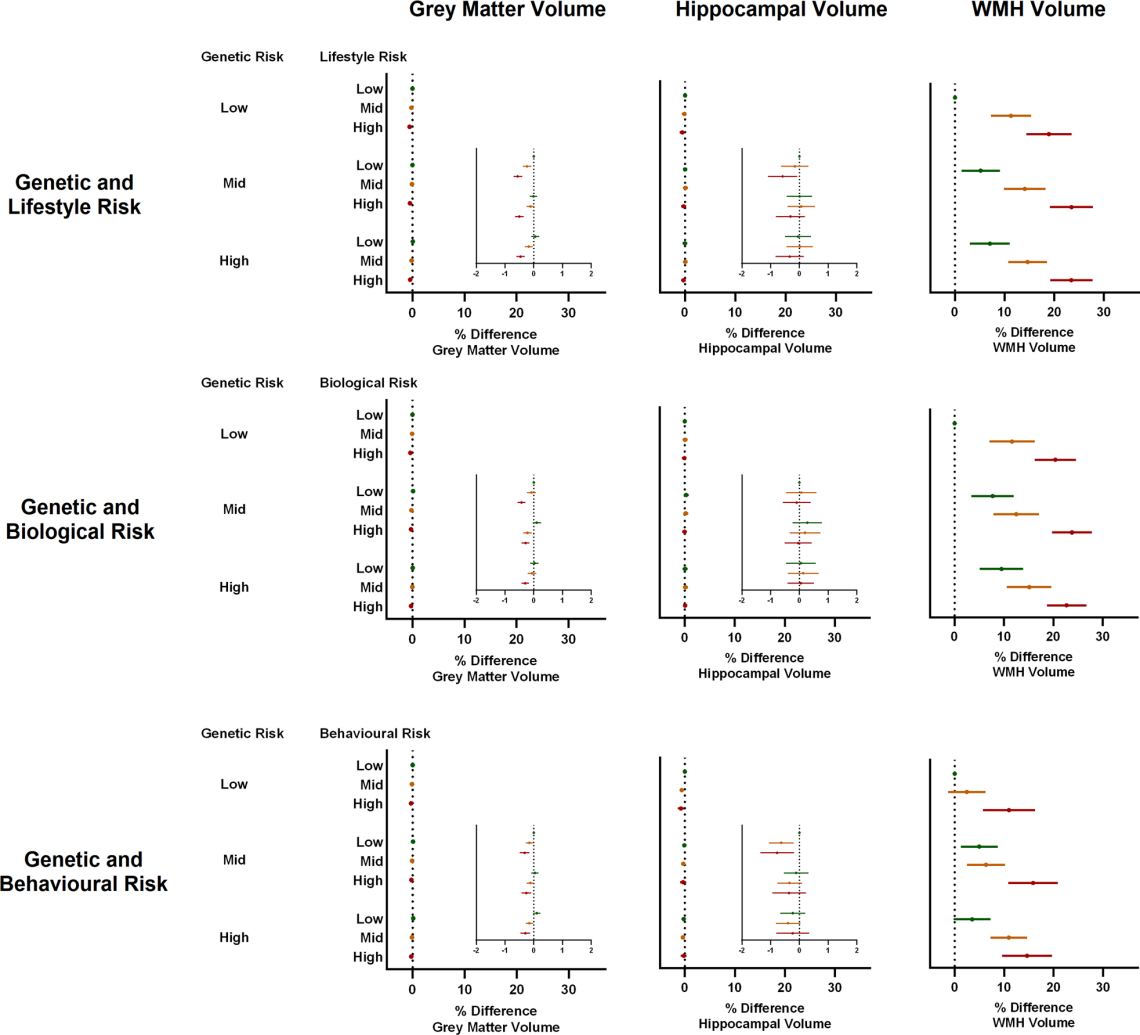
**Forest plots showing percent difference in neuroimaging phenotypes for combined genetic and lifestyle risk for coronary artery disease.** Multivariable regression models adjusted for age, sex, highest education level, socioeconomic status, baseline medication use, total brain volume (not white matter hyperintensities [WMHs]), and childhood body size. Data are expressed as percent difference in geometric mean and 95% CI compared with the reference category of combined low genetic and lifestyle risk. Low, medium, and high categories of genetic risk refer to tertiles of polygenic risk score. Lifestyle risk refers to categories of an overall lifestyle risk score comprising body mass index, blood pressure, lipid level, blood glucose level, diet, physical activity, smoking status, and sleep duration. Biological risk refers to categories of a risk score comprising body mass index, blood pressure, lipid level, and blood glucose level. Behavioral risk refers to categories of a risk score comprising diet, physical activity, smoking status, and sleep duration.

WMH volumes were associated with both genetic and lifestyle risks for CAD, with an increase in WMH volume of ≈3% and ≈8% observed for every SD increase in PRS and LRS, respectively (*P*<0.001 for both; Table 4). When combining genetic and lifestyle risks, individuals with risk scores in the top tertile for both PRS and LRS were found to have WMH volumes ≈25% higher than those with low levels of each (Figure 3). No associations were seen between the CAD PRS and either gray matter or hippocampal volumes (Table 4). There was some evidence of an association between the LRS and these same outcomes, but the absolute changes were clinically negligible (≈0.2% decrease in both volumes per SD increase in LRS; Table 4).

**Table 4.**
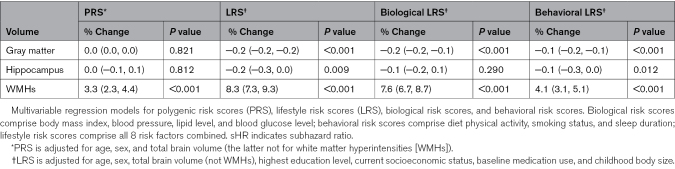
Associations Between Genetic and Lifestyle Risks for Coronary Artery Disease and Subclinical Neuroimaging Phenotypes

### Sensitivity Analyses

The rerunning of statistical models as complete case analyses had little bearing on findings (Table S2). Similar results were also obtained after excluding opposing dementia diagnoses as outcomes in models (Table S2). Separation of the LRS into biological and behavioral subscores identified positive associations between both biological and behavioral risk scores with current WMH burden and future risk of vascular dementia (Tables 2 and 3). Behavioral risk scores showed no associations with AD, whereas biological risk scores showed a seemingly paradoxical relationship with both Alzheimer’s and all-cause dementia. Further separation of the biological risk score into its individual components suggested that a strong negative association between BMI and AD was the most likely factor underlying this phenomenon (sHR, 0.75 [0.68, 0.81] for obesity versus normal weight; *P*<0.001; Table S3).

## Discussion

In this large UK-based longitudinal population study followed for up to 14 years, we provide 4 novel and important findings linking heart and brain health. First, we show that individuals who are genetically predisposed to developing CAD also face an increased risk of developing dementia in later life, suggesting that common pathogenic pathways may underlie the development of both diseases. Second, we observed that this risk is reduced in those demonstrating good cardiovascular health in the years preceding diagnosis, regardless of underlying genetic predisposition for CAD. Third, we show that this lifestyle-related risk reduction appears to be driven almost solely by a reduced incidence of vascular dementia cases rather than through any association with AD. Fourth, in a subset of individuals free from dementia at time of assessment, we provide evidence for early signs of vascular damage (ie, WMH burden) that broadly mirror patterns observed for progression to vascular dementia.

Associations between genetic variation and dementia risk have been extensively studied in recent years, but these have almost exclusively been for AD, the most common underlying cause of dementia.^7,8,34^ To our knowledge, no study to date has addressed the impact that a genetic predisposition to atherosclerotic vascular disease may also have on future vascular dementia risk. Given well-established links in the literature between heart and brain health,^35^ alongside previous evidence for reductions in cognitive^36^ and brain^37^ reserve in individuals at high genetic risk for coronary heart disease, we hypothesized that genetic variants associated with an increased risk for CAD may also increase the risk of dementia diagnoses caused predominantly by an underlying vascular pathology in those surviving to old age. Our findings support this hypothesis, demonstrating an ≈20% increased risk of all-cause dementia when comparing individuals in the top versus bottom tertile for a genomewide CAD PRS. By using the statistical power afforded by the large number of incident dementia cases available in the UK Biobank to separate these diagnoses into Alzheimer’s and vascular dementia cases, we found that this observed increase in risk was roughly double that for individuals diagnosed with the less common form of vascular dementia (≈33%) compared with AD (≈18%). Together, these findings suggest that shared genetic factors may underlie the development of both CAD and dementia cases that possess an underlying vascular pathology, perhaps through joint detrimental effects on the vasculature that affect perfusion within both the heart and brain.

Given that an individual’s genotype is fixed at conception, the question of whether this increased genetic risk is deterministic, or is instead mediated through other downstream pathways potentially amenable to modification, is an important consideration for population health and prevention strategies. It is now well-established that much of the genetic risk for CAD can be offset through the adoption of healthy lifestyle behaviors,^38–40^ and recent years have seen an increased focus on whether the same may be true for dementia.^7,8,35^ This interest has been spurred on in large part by the 2024 Lancet Commission Report on Dementia, in which it has been estimated that up to 45% of all dementias may be preventable by addressing 14 potentially modifiable risk factors with well-established links to heart disease.^41^ In support of these claims, we found that risk for incident all-cause dementia over 14 years of follow-up was lower in those with better cardiovascular health scores at their baseline assessment, regardless of underlying genetic risk. Furthermore, this reduction appeared to be driven almost exclusively through a reduced incidence of vascular dementia diagnoses, as well as being broadly mirrored by the magnitude of WMH burden measured in a subset of participants undergoing neuroimaging assessments.

Together, these findings have several potentially important implications. First, with the heritability of AD predicted to be in the range of 70% to 80%,^42^ opportunities to modify dementia outcomes arising from AD-related pathologies may be challenging except through pharmacological means. This is supported by findings from the current study, in which our observed associations linking healthier lifestyles to lower dementia risk were found to be essentially absent for AD. Furthermore, when separating lifestyle risk scores into biological and behavioral components, we observed evidence of a seemingly paradoxical negative relationship between increased biological risk and a reduced risk of dementia, with this effect again seemingly driven by the more common Alzheimer’s form of the disease. These findings may plausibly be explained by 2 scenarios, neither of which is mutually exclusive. First, the possibility exists that lifestyle prevention strategies, at least in the mid- to late-life period studied here, may not be as effective at reducing dementia risk in individuals with a strong genetic underpinning for Alzheimer’s-related disease. Support for this hypothesis has been demonstrated in a range of previous studies investigating associations between lifestyle risk and dementia when stratifying either by *APOE4* carrier status^43,44^ or wider polygenic risk for AD.^8^ Together with the current findings, these studies suggest that lifestyle-related benefits observed in studies of all-cause dementia may instead largely stem from the prevention of vascular-related or mixed dementia pathologies, rather than Alzheimer’s dementia per se. Indeed, the improved management of cardiovascular disease in recent decades is hypothesized to provide the most likely explanation for the unexpected reductions in age-standardized dementia rates that have recently been observed in the developed world.^45^ Alternatively, differences between Alzheimer’s and vascular dementia findings may stem from the different pathogeneses and developmental time scales of each of these diseases. Whereas both biological and behavioral risk factors were associated with an increased risk of WMH burden and vascular dementia in the current study, our biological risk score was unexpectedly found to negatively associate with Alzheimer’s (and therefore all-cause) dementia. Further sensitivity analyses identified a strong negative relationship between BMI and future Alzheimer’s diagnoses as the primary underlying reason for this association, a phenomenon that has previously been documented in this same cohort and, through genetic techniques, been ascribed to a reverse causal effect (whereby weight loss occurs during the long preclinical phase of Alzheimer’s dementia rather than the other way around).^46^ These findings highlight the care that must be taken when using composite exposures (such as risk scores) or composite outcomes (such as all-cause dementia) to link heart and brain health, because the distinct pathogeneses of both may mask or distort true associations that can only be identified by more granular analyses. In the current study, it is plausible for the long prodromal phase of Alzheimer’s dementia to result in changes in biological risk factors (eg, BMI) in some individuals, whereas this same risk factor simultaneously increases risk for the vascular form of the disease (which may often have a faster onset and follow an acute cardiovascular event such as a stroke) in others.

In support of this latter point, when assessing underlying neuroimaging phenotypes in a subset of the cohort recalled for brain magnetic resonance imaging, we observed significantly higher volumes of WMHs (a well-established marker of vascular damage) in individuals at increased genetic and lifestyle risk for CAD. These associations appeared to be additive, such that those at high risk for CAD through both genes and lifestyle were found to have WMH volumes ≈25% higher than those at low risk. In contrast, cortical gray matter volumes and hippocampal volumes, 2 imaging metrics with well-established links to AD, showed no association with our CAD PRS and demonstrated only negligible associations with our lifestyle risk score.

Future work is required to investigate these biological pathways and to determine how they link genetic risk for CAD to late-life dementia incidence. Recent work using Mendelian randomization techniques to investigate a potential causal connection between these diseases has found little evidence connecting manifest CAD to brain health,^47^ suggesting that overt ischemic heart disease per se is unlikely to be the direct causal mechanism influencing dementia risk. The use of a genomewide PRS in the current study instead suggests that both CAD and vascular dementia may possess a shared underlying genetic architecture that simultaneously increases risk of both diseases. This risk could manifest through the downstream mediation of established biological risk factors lying on the causal pathway between genes and both diseases (ie, vertical pleiotropy), as has been suggested in one recent study for BP.^48^ Alternatively, associations could represent evidence of gene–environment correlations (in which genes influence an individual’s exposure to adverse social environments that themselves influence risk), or horizontal pleiotropy (in which CAD genes influence dementia risk through other traits independent of CAD per se). Regardless of the underlying mechanisms, however, our findings demonstrate that genetic risk for CAD also increases risk of predominantly vascular dementia, particularly in those who do not adhere to well-established cardiovascular health guidelines earlier in their life course.

This study has limitations inherent to many population studies. First, this study is not designed to test for causal relationships between any of the genetic or lifestyle factors studied here and risk of dementia. Instead, it seeks to address the potential for a shared genetic architecture underlying both conditions and investigate whether this risk may be attenuated in the presence of healthy lifestyle choices. Second, participants reporting non-White ancestry were excluded to minimize residual confounding because of population stratification. Future studies addressing this research question in diverse ancestries are therefore warranted. Third, information about some lifestyle factors were obtained by self-report and biases may inevitably occur. Fourth, healthy lifestyle factors were not randomly assigned, unlike genetic makeup. Thus, despite efforts to adjust for potentially confounding factors, it is possible that other external factors may have been responsible for the relationship between lifestyle risk and dementia. Fifth, dementia diagnoses were obtained from electronic health records. This form of dementia ascertainment may be at risk of misclassification bias or lack sensitivity because of some cases of dementia not being recorded in hospital inpatient records or death registries. Although it is not possible to calculate sensitivity in UK Biobank (because to do so would require the true number of people with dementia in a population to be known [ie, including those with dementia who are undiagnosed and therefore not known to health care services]), recent work has been conducted to ascertain the positive predictive value afforded by the algorithm. This has reported positive predictive values of ≈83%, 71%, and 44% for all-cause, Alzheimer’s, and vascular dementia, respectively, suggesting adequate performance of these measures in this large population cohort.^21^

### Conclusions

In this large prospective UK population-based cohort, we provide evidence for an association between an elevated genetic risk for CAD and an increased risk of dementia. This risk was reduced in individuals maintaining optimal cardiovascular health in the years preceding diagnosis, specifically in individuals at risk of progression to vascular dementia. Overall, our findings indicate that, regardless of underlying genetic risk for CAD, lifestyle modifications targeting cardiovascular risk may also lower risk of dementia in older age, especially in diagnoses with an underlying vascular component.

## Article Information

### Acknowledgments

This research was conducted using the UK Biobank Resource under application No. 71702 and includes data provided by patients and collected by the National Health Service as part of their care and support.

### Sources of Funding

Dr Garfield is supported by a Diabetes Research and Wellness Foundation Professor David Matthews non-clinical fellowship (SCA/01/NCF/22). Dr Chiesa is supported by an Alzheimer’s Research UK David Carr fellowship (ARUK-RF2021B-006).

### Disclosures

None.

### Supplemental Material

Tables S1–S3

Figure S1
